# A Robust and High-Dimensional Clustering Algorithm Based on Feature Weight and Entropy

**DOI:** 10.3390/e25030510

**Published:** 2023-03-16

**Authors:** Xinzhi Du

**Affiliations:** School of Computer Science and Technology, Anhui University of Technology, Ma’anshan 243032, China; duxinzhi@ahut.edu.cn

**Keywords:** fuzzy clustering, high-dimensional data, feature weights, entropy weights, non-Euclidean distance

## Abstract

Since the Fuzzy C-Means algorithm is incapable of considering the influence of different features and exponential constraints on high-dimensional and complex data, a fuzzy clustering algorithm based on non-Euclidean distance combining feature weights and entropy weights is proposed. The proposed algorithm is based on the Fuzzy C-Means soft clustering algorithm to deal with high-dimensional and complex data. The objective function of the new algorithm is modified with the help of two different entropy terms and a non-Euclidean way of computing the distance. The distance calculation formula enhances the efficiency of extracting the contribution of different features. The first entropy term helps to minimize the clusters’ dispersion and maximize the negative entropy to control the clustering process, which also promotes the association between the samples. The second entropy term helps to control the weights of features since different features have different weights in the clustering process. Experiments on real-world datasets indicate that the proposed algorithm gives better clustering results than other algorithms. The experiments demonstrate the proposed algorithm’s robustness by analyzing the parameters’ sensitivity and comparing the computational distance formulas. In summary, the improved algorithm improves classification performance under noisy interference and high-dimensional datasets, increases computational efficiency, performs well in real-world high-dimensional datasets, and encourages the development of robust noise-resistant high-dimensional fuzzy clustering algorithms.

## 1. Introduction

In the field of machine learning and data mining, research on clustering has always attracted extensive attention [1,2,3,4]. Clustering methods are primarily categorized as partition-based, density-based, and hierarchical clustering methods [5,6,7]. Partition-based clustering methods classify different samples on the basis of features. In general, density-based clustering methods classify samples on the basis of the number of samples at each location. The samples are considered related (contained and included) and classified by the hierarchy of different samples in hierarchical clustering methods. Hierarchical clustering is suitable for small datasets but not large datasets. Partition-based clustering is one of the most used clustering methods for large dataset. Density-based clustering divides data points into high-density and low-density regions and is suitable for large datasets. In recent years, three-way soft clustering has been a new direction in clustering research, which attributes samples in the positive region as belonging to the cluster, samples in the boundary region as partially belonging to the cluster, and samples in the negative region as not belonging to the cluster [8,9,10]. Clustering divides the objects in the set into different clusters based on a certain standard (distance) to increase the intra-cluster similarity and reduce the inter-cluster similarity. Clustering algorithms are divided into soft and hard clustering on the basis of clustering research. Hard clustering specifies that a sample can be divided into only one cluster, while soft clustering allows a sample to be divided into different clusters. For two primary clustering methods, K-means and FCM clustering methods are the most widely used [11,12]. These two algorithms have driven the research on clustering, and much research has been performed to improve them. However, noise and initial cluster centers often influence the clustering results. Moreover, the clustering result is often significantly reduced when the clustering algorithm faces high-dimensional and complex data. A good clustering algorithm is supposed to be high-performance, robust, and scalable.

To resolve these difficulties, several improved clustering algorithms have been proposed [13,14,15,16]. The K-means method divides each sample into a specific cluster. However, these clusters often have overlapping and fuzzy divisions in practical applications. To solve the uncertain data objects, soft clustering algorithms are introduced. Bezdek [17] introduced the fuzzy set theory into K-means and proposed the FCM algorithm, which uses the membership function to verify the membership relationship between objects and clusters. Furthermore, it has demonstrated efficient performance in different applications [18,19]. However, the degree of the membership function does not always correspond to the cluster to which it belongs. The FCM algorithm is partition-based. The advantage of the partition-based clustering method is that the convergence is fast, and the disadvantage is that it requires that the number of clusters can be reasonably estimated and that the choice of initial centers and noise can have a significant impact on the clustering results. In these two traditional methods, all features are given the same weight and are easily influenced by noise [20,21]. These methods are also susceptible to random initial cluster centers, and poor initialization will likely result in local optimal solution generation [22,23]. In the face of high-dimensional and complex data, high-dimensional data are usually very sparse in space, and the sample size always seems very small compared with the dimensionality of the space. The features of clusters are not obvious. Traditional clustering algorithms cannot guarantee robustness; hence, it is extremely important to fully use the features’ properties.

To address these issues, it is critical to remember that different weights should be assigned to different features. In previous studies, the weights corresponding to the features were assigned in one of two ways. The first method is to assign weights to the features on a global scale. Throughout the process, a specific feature is given only one weight. The second method is to assign features local weights, which means that features in a dataset have different weights in different clusters. Numerous studies have demonstrated that the second method outperforms global weighting [24]. Therefore, the SCAD algorithm considers different feature weights in the different clusters and simultaneously clusters the data with feature discrimination [25]. However, the conventional FCM algorithms, including improved algorithms, constrain related variables through exponential regularization, which may lead to consistent results and low precision when dealing with sparse and noisy data (the denominator is 0). Entropy is proposed to go for better-constrained features in the clustering process. Entropy-Weighting K-Means is particularly prominent method among local weight-based and entropy-weight algorithms [26]. EWKM pioneered the form of entropy weight and applied it to membership to better constrain the objective function. The algorithm introduced the form of entropy weight in response to this defect to incentivize more features that contribute to identifying clusters. The newly introduced approach is more efficient in dealing with various data types, particularly high-dimensional sparse data. Entropy weights can be used to assign relevant feature weights to a cluster, whereas fuzzy partitions can help identify the best partitions. This method, however, ignores the limitations of Euclidean distance and may result in inconsistent classification [24,27]. Moreover, the weights of this algorithm are very dependent on the initial clustering centers and are sensitive to changes related to the centers. If the initial cluster center varies, the algorithm will not be robust and will not converge. Therefore, improving the clustering algorithm should focus on these aspects. To address the effect of noise and features with different weights in different clusters, certainly improved algorithms for classification criteria based on non-Euclidean distances have been proposed [28,29,30]. Some algorithms using entropy weights have also been proposed in unsupervised clustering studies. Both Entropy K-Means [31] and U-K-Means [32] algorithms use feature weights and entropy weights in unsupervised learning. However, the distance formula used in their objective functions is Euclidean distance, which does not perform well in the face of high-dimensional and noisy data. This can eventually lead to noise and irrelevant features influencing the overall clustering results. In recent years, there has been extensive interest in research on feature weights and entropy weights for fuzzy clustering. However, these algorithms frequently only consider the Euclidean distance and do not constrain features or use fuzzy partitioning [33,34,35].

This paper proposes an advanced FCM algorithm to handle high-dimensional sparse and noisy data to solve the flaws mentioned above. The proposed algorithm’s new findings use entropy control features and partitions, and adding non-Euclidean distance division eliminates noise interference. Moreover, the entropy weight is introduced to the membership and weight variables to enhance its efficiency in processing different datasets. In improving clustering in the face of high-dimensional and complex data, the intra-cluster dispersion is minimized, and the negative weight entropy is maximized to motivate features to help identify clusters. Furthermore, the proposed method updates the membership degrees of samples in different clusters and the feature weights in different clusters during each iteration so that the objective function converges rapidly. This algorithm avoids this issue by efficiently handling high-dimensional data and noise with feature weights and non-Euclidean distance formulas. Furthermore, entropy weights are used to constrain variables, which can be more advantageous than exponential constraints in some cases. Extensive experimental results on real datasets suggest that the proposed algorithm performs better in clustering. The proposed algorithms on high-dimensional and complex datasets also exhibit high performance. Furthermore, the algorithm exhibits robustness and stability in the experiment.

The remainder of the paper comprises the following sections: Section 2 introduces several of the most classic clustering methods. In Section 3, the proposed algorithm, its convergence proof, and its complexity analysis are provided. The performance of the proposed algorithm and other clustering algorithms is compared and evaluated using different clustering metrics in Section 4. Lastly, Section 5 presents a summary of the paper.

## 2. Related Work

### 2.1. The K-Means Algorithms

For a given dataset $X_{N\times M}$, N denotes the number of samples, M denotes the number of features, K represents the number of clusters, $x_{ij}$ denotes the jth feature in the ith sample, $c_{ij}$ denotes the cluster center of the jth feature in the ith cluster, and $u_{ij}$ denotes whether the ith sample belongs to the kth cluster. ${\left(x_{ij}-c_{lj}\right)}^{2}$ is the Euclidean distance between the ith sample and the jth cluster at the lth feature. The objective function can be defined as

(1) $$P(U,C)=\sum_{i=1}^{N}\sum_{j=1}^{M}\sum_{l=1}^{K}u_{il}{\left(x_{il}-c_{lj}\right)}^{2},$$

subject to

(2) $$\sum_{l=1}^{K}u_{il}=1.$$

The K-means can be minimized by continuously iterating the following equations:

(3) $$u_{il}=\left\{ \begin{array}{l} 1,\sum_{j=1}^{M}{\left(x_{ij}-z_{lj}\right)}^{2}\leq \sum_{j=1}^{M}{\left(x_{ij}-z_{tj}\right)}^{2},1\leq t\leq K,\\ 0,else. \end{array}\right.$$

(4) $$c_{lj}=\frac{\sum_{i=1}^{N}u_{il}x_{ij}}{\sum_{i=1}^{N}u_{il}},$$

where $z_{lj}$ denotes the value of the jth feature in the lth clustering center, and t denotes all the clustering centers in the clustering process.

The proposed K-means algorithm has significantly contributed to the study of clustering. However, when noise is encountered, the noise dimension is also considered in the results while computing the distance between the samples, leading to decreased clustering accuracy. Furthermore, as the dimensionality of the dataset increases, so does the number of outlier points and the dispersion between samples, affecting the clustering center and changes the clustering results. K-means clustering is often inefficient when dealing with high-dimensional sparse and noisy data. Moreover, it is susceptible to the initial clustering center.

### 2.2. The Weighting K-Means Algorithms

WK-Means generalizes the K-means and introduces a new algorithm to solve the noise data [30] skillfully. It considers that different features must have different weights so that the effect of the noise dimension can be ignored as much as possible when evaluating the distance between samples. Therefore, noise far from the cluster centroid is given a smaller weight and has less influence on the cluster centroid. Therefore, the clustering accuracy is improved. The objective function of WK-Means is as follows:

(5) $$P(U,C,W)=\sum_{i=1}^{N}\sum_{j=1}^{M}\sum_{l=1}^{K}u_{il}w_{j}{}^{\beta }{\left(x_{il}-c_{lj}\right)}^{2},$$

subject to

(6) $$\left\{ \begin{array}{l} \sum_{l=1}^{K}u_{il}=1,\\ \sum_{j=1}^{M}w_{j}=1. \end{array}\right.$$

The WK-means can be minimized by continuously iterating the following equations:

(7) $$u_{il}=\left\{ \begin{array}{l} 1,w_{j}{}^{\beta }\sum_{j=1}^{M}(x_{ij}-c_{lj})\leq w_{j}{}^{\beta }\sum_{j=1}^{M}{\left(x_{ij}-c_{tj}\right)}^{2},1\leq t\leq K,\\ 0,else. \end{array}\right.$$

(8) $$c_{lj}=\frac{\sum_{i=1}^{N}u_{il}x_{ij}}{\sum_{i=1}^{N}u_{il}}.$$

(9) $$w_{j}=\frac{1}{\sum_{t=1}^{M}{\left[\frac{D_{j}}{D_{t}}\right]}^{\frac{1}{\beta -1}}},\beta >1or\beta \leq 0,$$

where

(10) $$D_{j}=\sum_{l=1}^{K}\sum_{i=1}^{N}u_{il}{\left(x_{ij}-c_{lj}\right)}^{2}.$$

In Equation (5), $w_{ij}$ indicates whether the ith sample belongs to the kth cluster, $w_{j}$ denotes the weight of the jth feature, and β represents a fuzzy constant, usually taken as 2.

Although the WK-means algorithm was the first to use weights, targeting global weights performs poorly in some data, and hard clustering may result in results being fixed. It can be deduced that the weight difference between features is not always visible when the dataset is high-dimensional. The FCM algorithm based on fuzzy ideas significantly reduces the singularity of clustering and has several advantages in terms of high-latitude data.

### 2.3. Fuzzy C-Means Algorithm

As the most representative soft clustering method, FCM defines a new method for dividing the clusters. Each sample has a different degree of membership for each cluster, and its cluster assignment is determined by the degree of membership. The objective function can be defined as

(11) $$P(U,C)=\sum_{i=1}^{N}\sum_{j=1}^{M}\sum_{l=1}^{K}u_{il}{}^{m}{\left(x_{il}-c_{lj}\right)}^{2},$$

subject to

(12) $$\sum_{l=1}^{K}u_{il}=1.$$

The FCM is minimized by continuously iterating the following equations:

(13) $$c_{i}=\frac{\sum_{i=1}^{N}u_{ij}{}^{m}x_{ij}}{\sum_{i=1}^{N}u_{ij}{}^{m}}.$$

(14) $$u_{ij}={\left[\sum_{t=1}^{K}{\left(\frac{{\left(x_{ij}-c_{lj}\right)}^{2}}{{\left(x_{ij}-c_{tj}\right)}^{2}}\right)}^{\frac{2}{m-1}}\right]}^{-1},$$

where $u_{ij}$ represents the degree of the ith data’s membership to the jth cluster, and m is the number of fuzzy factors.

Although the FCM algorithm is the most representative soft clustering algorithm, it still has numerous flaws and shortcomings. For example, the algorithm only counts the “nearest” neighbor samples, and the number of samples in a category is very large. The algorithm prioritizes that sample, which affects the clustering results. Moreover, fuzzy factors as exponential forms often do not constrain the features well, leading to the fact that it tends to perform much worse when combined with the Euclidean distance formula.

## 3. The Proposed Algorithm

In this section, a novel Fuzzy-C-Means-based entropy weighting algorithm is proposed. Motivated by the shortcomings of traditional Fuzzy-C-Means clustering algorithm, a new algorithm is presented that includes local feature weighting, the use of entropy weights acting on features, and the degree of membership to improve clustering’s sensitivity and accuracy to random class centers. Furthermore, because the Euclidean distance is susceptible to noise and outliers [29], a non-Euclidean distance is introduced. The new distance formula makes the algorithm more robust and fully uses the dataset’s features to get the clustering result. The objective function can be defined as

(15) $$F(U,C,W)=\sum_{i=1}^{N}\sum_{j=1}^{K}\sum_{l=1}^{M}u_{ij}w_{jl}(1-\exp(-\delta_{l}{\left(x_{il}-c_{jl}\right)}^{2})+\lambda \sum_{i=1}^{N}\sum_{j=1}^{K}u_{ij}\log u_{ij}+\gamma \sum_{j=1}^{K}\sum_{l=1}^{M}w_{jl}\log w_{jl}.$$

In Equation (15), $U=[u_{ij}]$ is an N by K matrix, in which $u_{ij}$ denotes the degree of the ith sample’s membership to the center of the jth cluster; $C=[c_{jl}]$ is a K by M matrix, where $c_{jl}$ represents the center of the jth cluster and is defined by $u_{ij}$. Moreover, $W=[w_{jl}]$ is a K by M matrix, where $w_{jl}$ denotes the weight of the lth feature in the jth cluster. U is the membership matrix of each sample to cluster, containing N samples and K clusters. C is the feature center matrix of each cluster, containing K clusters and M features. W is the feature weight matrix of each sample to cluster, containing K clusters and M features. The term $\left(1-\exp(-\delta_{{}_{l}}{\left(x_{il}-c_{jl}\right)}^{2}\right)$ denotes a non-Euclidean distance metric between the ith sample and the jth cluster in the lth feature and is defined as follows:

(16) $$\delta_{l}=\frac{1}{{\mathrm{var}}_{l}},{\mathrm{var}}_{l}=\sum_{i=1}^{N}\frac{{\left(x_{ij}-\bar{x_{l}}\right)}^{2}}{N},\bar{x_{l}}=\sum_{i=1}^{N}\frac{x_{il}}{N},$$

where $\delta_{l}$ denotes the inverse of the variance of the lth feature of the data,

subject to

(17) $$\left\{ \begin{array}{l} \sum_{j=1}^{K}u_{ij}=1,u_{ij}\in (0,1],1\leq i\leq N,\\ \sum_{l=1}^{M}w_{jl}=1,w_{jl}\in (0,1],1\leq j\leq K. \end{array}\right.$$

Minimizing F in Equation (15) with the constraints forms a class of constrained nonlinear optimization problems. The usual approach toward optimization of F is to introduce partial optimization for U, C, and W. First, U and C are fixed, and the reduced F is minimized with respect to W. Next, U and W are fixed, and the reduced F is minimized with respect to C. Followed by this, W and C are fixed, and the reduced F is minimized to solve U. After the results are calculated iteratively, the solution can be drawn.

The Lagrange multiplier technique is used to solve the following unconstrained minimization problem:

(18) $$\begin{array}{l} F(U,C,W)=\sum_{i=1}^{N}\sum_{j=1}^{K}\sum_{l=1}^{M}u_{ij}w_{jl}(1-\exp(-\delta_{l}{\left(x_{il}-c_{jl}\right)}^{2})+\lambda \sum_{i=1}^{N}\sum_{j=1}^{K}u_{ij}\log u_{ij}+\gamma \sum_{j=1}^{K}\sum_{l=1}^{M}w_{jl}\log w_{jl}\\ -\alpha (\sum_{j=1}^{K}u_{ij}-1)-\beta (\sum_{l=1}^{M}w_{jl}-1), \end{array}$$

where α and β are Lagrange multipliers. By setting the gradient of F with respect to α, β, $u_{ij}$, $c_{jl}$, and $w_{jl}$ to zero,

(19) $$\frac{\partial F}{\partial \alpha }=-(\sum_{j=1}^{K}u_{ij}-1)=0.$$

(20) $$\frac{\partial F}{\partial \beta }=-(\sum_{l=1}^{M}w_{jl}-1)=0.$$

(21) $$\frac{\partial F}{\partial u_{ij}}=\sum_{l=1}^{M}w_{jl}(1-\exp(-\delta_{l}{\left(x_{il}-c_{jl}\right)}^{2})+\lambda (1+\log u_{ij})-\alpha =0.$$

(22) $$\frac{\partial F}{\partial w_{jl}}=\sum_{i=1}^{N}u_{ij}(1-\exp(-\delta_{l}{\left(x_{il}-c_{jl}\right)}^{2})+\gamma (1+\log w_{jl})-\beta =0.$$

From Equations (21) and (22),

(23) $$u_{ij}=\exp(\frac{\alpha }{\lambda })\exp(\frac{-D_{jl}}{\lambda })\exp(-1),$$

(24) $$w_{jl}=\exp(\frac{\beta }{\gamma })\exp(\frac{-{D^{\prime }}_{ij}}{\gamma })\exp(-1),$$

where

(25) $$D_{ij}=\sum_{l=1}^{M}w_{jl}(1-\exp(-\delta_{l}{\left(x_{il}-c_{jl}\right)}^{2}).$$

(26) $${D^{\prime }}_{ij}=\sum_{l=1}^{M}u_{ij}(1-\exp(-\delta_{l}{\left(x_{il}-c_{jl}\right)}^{2}).$$

From Equations (19) and (23),

(27) $$\sum_{t=1}^{K}u_{it}=1=\sum_{t=1}^{K}\exp(\frac{\alpha }{\lambda })\exp(\frac{-D_{tl}}{\lambda })\exp(-1),$$

where it follows that

(28) $$\exp(\frac{\alpha }{\lambda })=\frac{1}{\sum_{t=1}^{K}\exp(\frac{-D_{tl}}{\lambda })\exp(-1)},$$

which can be substituted into Equation (23),

(29) $$u_{ij}=\frac{\exp(\frac{-D_{jl}}{\lambda })}{\sum_{t=1}^{K}\exp(\frac{-D_{tl}}{\lambda })}.$$

From Equations (20) and (24),

(30) $$\sum_{t=1}^{M}w_{jt}=1=\sum_{t=1}^{M}\exp(\frac{\beta }{\gamma })\exp(\frac{-{D^{\prime }}_{tl}}{\gamma })\exp(-1),$$

where it follows that

(31) $$\exp(\frac{\beta }{\gamma })=\frac{1}{\sum_{t=1}^{M}\exp(\frac{-{D^{\prime }}_{tl}}{\gamma })\exp(-1)},$$

which can be substituted into Equation (24),

(32) $$w_{jl}=\frac{\exp(\frac{-{D^{\prime }}_{ij}}{\gamma })}{\sum_{t=1}^{M}\exp(\frac{-{D^{\prime }}_{it}}{\gamma })}.$$

For the clustering centers,

(33) $$\frac{\partial F}{\partial c_{jl}}=\frac{\partial \sum_{i=1}^{N}u_{ij}w_{jl}(1-\exp(-\delta_{l}{\left(x_{il}-c_{jl}\right)}^{2})}{\partial c_{jl}}=0,$$

where it follows that

(34) $$-\sum_{i=1}^{N}u_{ij}w_{jl}2\delta_{l}(x_{il}-c_{jl})\exp(-\delta_{l}{\left(x_{il}-c_{jl}\right)}^{2})=0,$$

which gives

(35) $$c_{jl}=\frac{\sum_{i=1}^{N}u_{ij}w_{jl}\delta_{l}\exp(-\delta_{l}{\left(x_{il}-c_{jl}\right)}^{2})x_{il}}{\sum_{i=1}^{N}u_{ij}w_{jl}\delta_{l}\exp(-\delta_{l}{\left(x_{il}-c_{jl}\right)}^{2})}.$$

It is evident that Equation (35) is independent of the parameters λ and γ. The interdependence of both terms promotes the detection of a better partition during the clustering process. The proposed algorithm minimizes Equation (15), using Equations (29), (32), and (35).

### 3.1. Parameter Selection

The values of λ and γ are essential for the proposed algorithm since they affect the significance of the second and third terms in Equation (15) relative to the first term. Initially, λ plays two roles in the clustering process; when λ is large, this results in a smaller value of $u_{ij}$ in Equation (29) and, thus, the second term has a more significant influence to minimize Equation (15). Therefore, it tries to assign more than one sample cluster to make the second term more negative while clustering. The membership entropy value becomes larger when the membership value $u_{ij}$ of a sample to all clusters is equal. After the position of the samples is fixed, all the clustering centers move to the same position for an enormous entropy value. Second, when λ is large, this results in an immense value of $u_{ij}$ in Equation (29). Therefore, the first term plays a key role in minimizing Equation (15). The local feature weights are controlled by γ. Since γ is positive, the value of $w_{jl}$ is inversely proportional to $\sum_{i=1}^{M}D_{ij}^{\prime }$. A smaller value of this term results in a larger $w_{jl}$. If γ is large, the third parameter controls the partitions, and all feature weights are assigned 1/M in different clusters.

Assuming that $F^{t}$ denotes the value of Equation (15) after the run, $F^{t+1}$ denotes the value after the next completion. The proposed algorithm is summarized below (Algorithm 1).
**Algorithm 1.** Proposed Clustering Algorithm.**Input:** Dataset $X_{N\times M}$, the number of clusters *K*, the values of parameters *λ* and γ. Randomly set *K* cluster centers, generate a set of initial weights, set *t* = 0, the maximum iteration is MAX, and local minimum $value=F^{t+1}-F^{t}$.**Output:** $U={\left[u_{ij}\right]}_{N\times K}$.**Repeat**1: Compute the non-Euclidean distance matrix $D_{N\times M}$.2: Update the partition matrix $U_{N\times K}$ using Equation (29).3: Update the weight matrix $W_{K\times M}$ using Equation (32).4: Update the cluster center matrix $C_{K\times M}$ using Equation (35).5: Until the objective function is less than or equal to the local minimum or reaches the maximum iterations.

### 3.2. Convergence Analysis

It is important to note that the proposed algorithm will converge in the iterations. It can be observed that different partition U occurs only once during the algorithm process. Therefore, it is assumed that $U^{i}=U^{j}$ where i≠j. It must be noted that given $U^{t}$. The minimizer $W^{t}$ should be calculated. For $U^{i}$ and $U^{j}$, the minimizers are $W^{i}$ and $W^{j}$, respectively. It is evident that $W^{i}=W^{j}$ since $U^{i}=U^{j}$. Using $U^{i}$, $W^{i}$, $U^{j}$, and $W^{j}$, the minimizers $C^{i}$ and $C^{j}$ can be calculated, respectively. It is obvious that $C^{i}=C^{j}$. Therefore, the following equation can be obtained:

(36) $$F(U^{i},C^{i},W^{i})=F(U^{j},C^{j},W^{j}).$$

However, the function F(⋅,⋅,⋅) monotonically decreases. Therefore, different partition U occurs only once during the algorithm process. $u_{ij}$ in Equation (29) can be calculated after taking the derivative of Equation (18) and setting it equal to zero. Therefore, $u_{ij}$ can be minimal or maximal. If the second partial derivative of Equation (18) is positive, it can prove that $u_{ij}$ defined by Equation (29) is a local minimum of Equation (18). The second partial derivative of Equation (18) with respect to $u_{ij}$ is

(37) $$\sum_{l=1}^{M}\frac{\lambda }{u_{ij}}.$$

Since $u_{ij}>0$, λ>0, it can prove that Equation (37) is positive. Therefore, K in Equation (29) is a local minimum of Equation (18). The proposed algorithm converges in a finite number of iterations.

### 3.3. Computational Complexity

As shown in Table 1, the computational complexity of the proposed algorithm is high when compared to other clustering algorithms. However, by adding new terms, the clustering performance is improved. The computational complexity of the algorithm is based on four update processes: updating the distance matrix D, cluster center matrix C, membership matrix U, and weight matrix W. The computational complexity of each process is equal to NKM. Each process executes independently; hence, the total computational complexity is 4NKM. Furthermore, N denotes the number of samples, K denotes the number of clusters, and M denotes the number of features. Each iteration updates D, C, U, and W using Equations (29), (32), and (35) and finally classifies the different samples according to the matrix U.

## 4. Experiments

In the experimental section, to test the performance of the proposed algorithm, the performance of other clustering algorithms is evaluated and compared on real-world datasets. These algorithms are the standard K-Means [11], the standard FCM [12], WK-Means [30], RLWHCM [28], SCAD [25], EWK-Means [31], and UK-Means [32]. These algorithms have specific standard parameters. If the parameter values are uniform, the influence of the parameters can be removed to observe the clustering results. Therefore, various parameters were equalized to avoid inconsistency in algorithm performance. In the experiments, the maximum number of restarts was set to 100. For λ=0.3 and γ=1.4, the clustering centroids were randomly selected from the original datasets. In practical applications, choosing the appropriate threshold value is a crucial issue. If the threshold is too small, the algorithm may converge very slowly or not even converge; if the threshold is too large, the algorithm may stop prematurely, resulting in less accurate clustering results. Therefore, experiments and adjustments are needed to determine an appropriate threshold size. Considering that the threshold value is not the same for different datasets, to get the best performance, we reduced from 0.1 to 0.00001 by 10 times in each case to get the best clustering result. After testing, $value=10^{-5}$ was set to get accurate clustering results for different datasets. Each algorithm was iterated 100 times, and the best result was recorded.

Seven real-world datasets from UCI [36] were used to assess the performance of the proposed approach and compare its results to other approaches. Furthermore, the text, face image, and biological datasets [37] highlight the proposed algorithm’s performance in high-dimensional and noisy datasets. These datasets are mentioned in Table 2. The best clustered values for each dataset in Table 3 and Table 4 are bolded.

### 4.1. Evaluation Indicators

The clustering accuracy is defined as

(38) $$ACC=\frac{\sum_{l=1}^{K}D_{l}}{N}.$$

In Equation (38), $D_{l}$ denotes the number of samples correctly classified into the lth cluster, and N represents the number of points in the dataset. A considerable value of ACC [38] suggests a better clustering performance.

Since the sample labels of the real-world dataset are known, RI [38] was used to evaluate the similarity between the clustering partitions and real partitions.

(39) $$RI=\frac{f_{1}+f_{3}}{f_{1}+f_{2}+f_{3}+f_{4}}.$$

In Equation (39), $f_{1}$ indicates the number of similar sample points belonging to a common partition, $f_{2}$ indicates the number of non-similar samples belonging to a common partition, $f_{3}$ indicates the number of non-similar samples in two separate partitions, and $f_{4}$ indicates the number of common samples belonging to two different partitions. Larger values suggest better classification results.

NMI is often used in clustering to compute the similarity between the clustering results and the real label of the dataset. The measurement method is

(40) $$NMI(A,B)=\frac{\sum_{i=1}^{I}\sum_{j=1}^{J}P(i,j)\log\frac{P(i,j)}{P(i)P(j)}}{\sqrt{H(A)H(B)}}.$$

In Equation (40), A and B are the two partitions in the dataset comprising clusters I and J, respectively. P(i) indicates the probability that a randomly selected sample is allocated to a cluster $A_{i}$, P(i,j) represents the probability that a sample belongs to both clusters $A_{i}$ and $B_{i}$, and H(A) is the entropy associated with all the probabilities P(i) in partition A [39]. A larger value of NMI leads to more consistent clustering results.

### 4.2. Clustering Results on Real-World Datasets

As demonstrated in Table 3 and Table 4, the clustering performance of the proposed algorithm in terms of ACC, RI, and NMI was much better on different real-world datasets than other clustering methods. The clustering results suggest that the proposed algorithm significantly improved the clustering performance and provided the best results in most real-world and txt datasets. It should also be noted that the proposed algorithm’s performance was not exceptional in the Wine dataset. After careful examination of this dataset, it was discovered that there were only three clusters in this dataset, and there was little variation in the values of the features. The value of a feature in different clusters may differ by as little as 0.1, which negatively impacts any clustering algorithm and leads to poor performance of entropy-weight terms and non-Euclidean distances. Most classical clustering algorithms had ACC and NMI values of nearly 0.5 in the high-dimensional datasets because the number of clusters was two, indicating that these algorithms failed due to high dimensionality and noise. The reason is that there are often many outliers in high-dimensional data, and the data distribution could be sparser. Traditional clustering algorithms have difficulty clustering these sparse data. As the dimensionality increases, the calculation based on distance makes the clustering centers progressively more complex and is affected by outliers leading to shifts. At the same time, because the different feature weights of different clusters are not considered, the feature of sparse high-dimensional data cannot be exploited, making the clustering results much less accurate. However, using the entropy constraint feature in the proposed algorithm improves the algorithm’s performance in three performance metrics. On the other hand, the clustering algorithm with exponential constraints equalizes sample membership degrees, resulting in an inaccurate ACC value of 0.5. The results also show that the proposed algorithm has the advantage of assisting in the detection of noise and the main classification features in large datasets. The clustering results of the proposed algorithm performed better in the Face Image Dataset and Biological Dataset. This shows that, with random initialization of clustering centers, our algorithm was better and more stable in handling high and noisy datasets. From the clustering results, we can find that the K-Means algorithm performed better in the face dataset because it did not introduce the feature weights, which could lead to unsatisfactory results for high-dimensional data and when the number of clusters is large. Furthermore, this demonstrates that the algorithm can be used for classification in both supervised and unsupervised learning.

Numerous points justify the performance of the proposed algorithm. First, this algorithm has good performance even with terrible initial centers, while the other algorithms are severely sensitive to initialization. Second, the introduction of entropy weighting allows different features to be well added to the clustering process. Third, the non-Euclidean distance makes it possible to encounter noisy and sparse data in the calculation and does not affect the clustering results.

**Figure 1:**
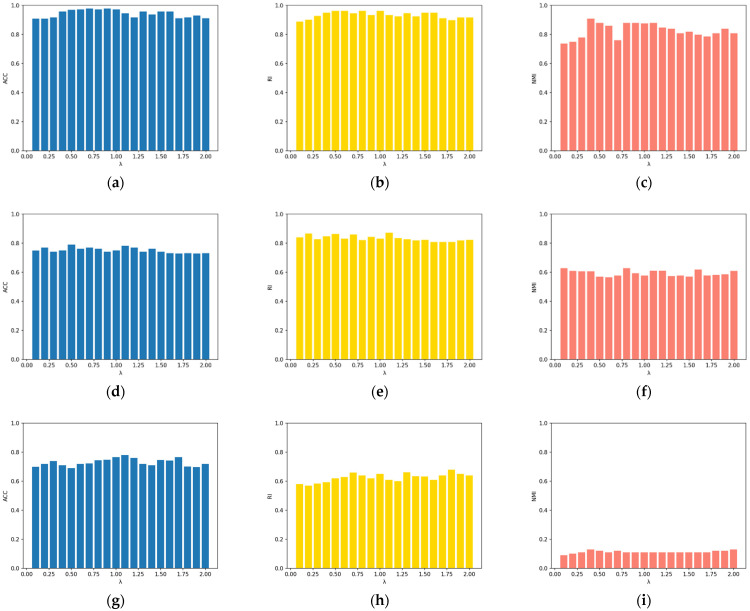
The sensitivity of λ when γ=0.8: (**a**) ACC of Iris dataset with γ=0.8; (**b**) RI of Iris dataset with γ=0.8; (**c**) NMI of Iris dataset with γ=0.8; (**d**) ACC of Zoo dataset with γ=0.8; (**e**) RI of Zoo dataset with γ=0.8; (**f**) NMI of Zoo dataset with γ=0.8; (**g**) ACC of PCMAC dataset with γ=0.8; (**h**) RI of PCMAC dataset with γ=0.8; (**i**) NMI of PCMAC dataset with γ=0.8.

**Figure 2:**
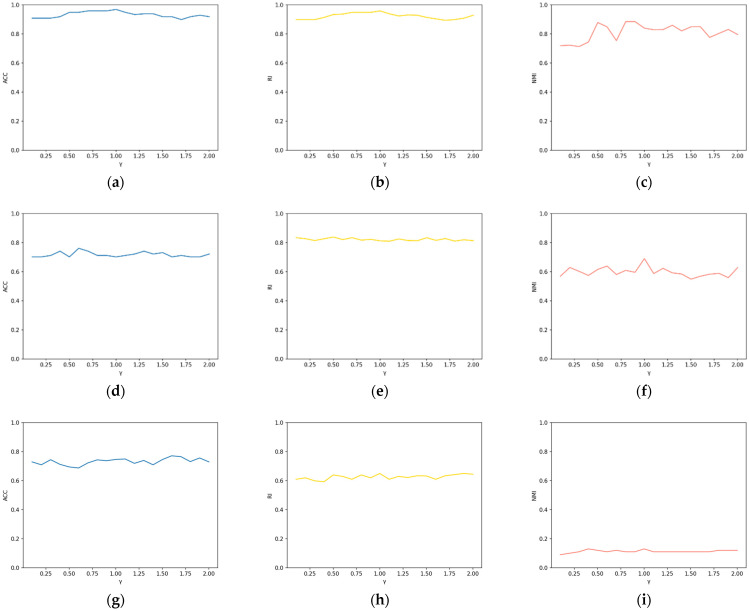
The sensitivity of γ when λ=0.3: (**a**) ACC of Iris dataset with λ=0.3; (**b**) RI of Iris dataset with λ=0.3; (**c**) NMI of Iris dataset with λ=0.3; (**d**) ACC of Zoo dataset with λ=0.3; (**e**) RI of Zoo dataset with λ=0.3; (**f**) NMI of Zoo dataset with λ=0.3; (**g**) ACC of PCMAC dataset with λ=0.3; (**h**) RI of PCMAC dataset with λ=0.3; (**i**) NMI of PCMAC dataset with λ=0.3.

**Figure 3:**
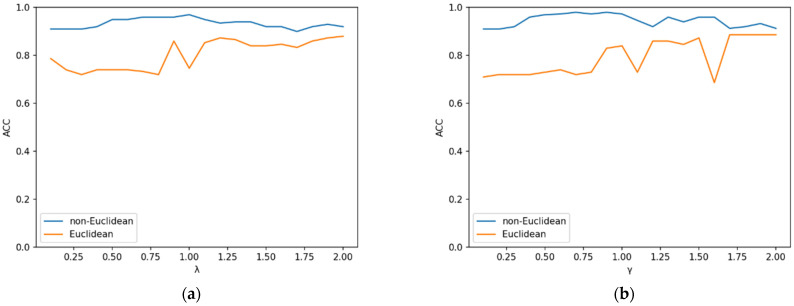
Comparison of Euclidean distance and non-Euclidean distance at ACC: (**a**) ACC of Iris dataset with γ=0.8; (**b**) ACC of ZOO dataset with λ=0.3.

The value range of the parameters was discussed in detail in the previous section. To further examine the sensitivity of λ and γ, the algorithm’s sensitivity was analyzed on the Iris and Zoo datasets. λ = 0.3 was fixed, increasing γ from 0 by 0.1 to 2 each time. The sensitivity of λ can be inferred from Figure 1. After that, γ = 0.8 was fixed, increasing λ from 0 by 0.1 to 2 each time. The sensitivity of γ can be observed in Figure 2. It can be observed from the figures that ACC, RI, and NMI did not fluctuate much when the two parameters were varied, which highlights the excellent performance and robustness of our algorithm. Higher values for the three metrics also directly reflect the proposed algorithm’s efficiency and robustness. The ACC changes of the proposed algorithm were analyzed on the basis of the non-Euclidean distance and the Euclidean distance tested on the Iris and Zoo datasets, to better demonstrate this advancement of the proposed non-Euclidean distance in computing sample point distances. Figure 3 shows that clustering results based on non-Euclidean distances were more accurate than those based on Euclidean distances. In the non-Euclidean distance, the average ACC values were 0.95 and 0.93, respectively, while in the Euclidean distance, the average ACC values were 0.79 and 0.80, respectively. The new distance formula increased the ACC of the algorithm by 18%, which also indicates the advantage of the newly proposed non-Euclidean distance in dealing with high-dimensional sparse and noisy data. Furthermore, the ACC’s variance of non-Euclidean distance was 0.025, while the ACC’s variance of Euclidean distance was 0.074, indicating that the non-Euclidean distance makes the algorithm more robust. To better distinguish the algorithms in the experiment, Table 5 reflects the usage conditions associated with the compared algorithms. The experimental results show that the proposed algorithm had high clustering accuracy in most real-world datasets from various domains. The sensitivity analysis of the two parameters could be determined, proving that the algorithm is robust and performs well. Furthermore, when comparing the Euclidean distance and the non-Euclidean distance on the objective function, we can find that the non-Euclidean distance was more accurate and stable in clustering results.

### 4.3. Discussion of Noise

**Figure 4:**
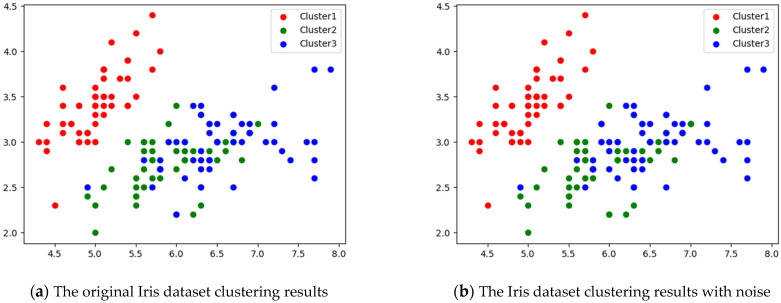
The effect of noise on the Iris dataset: (**a**) without noise; (**b**) with noise.

**Figure 5:**
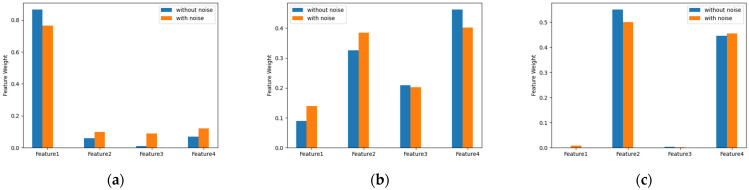
Weights of the features assigned in Iris dataset for (**a**) Cluster 1, (**b**) Cluster 2, and (**c**) Cluster 3.

To demonstrate the performance of the proposed algorithm under the influence of noise, a new experiment was designed for the Iris dataset. In the Iris dataset, uniformly distributed data from 0 to 1 were randomly assigned as new noise features. Furthermore, to compare the effect with and without noise, we compared experiments for the original dataset and the dataset with noise. As shown in Figure 4, the noise only slightly affected the clustering results. This shows that the performance of the proposed algorithm remained good even though the noise affected the dataset. Moreover, as shown in Figure 5, the noise did not influence the assignment of feature weights. This confirms the high accuracy and stability of the algorithm.

## 5. Conclusions

This paper proposed a new algorithm for classifying high-dimensional and noisy data on the basis of non-Euclidean distance, combining feature weights and entropy weights. In this approach, two different entropy terms are added to the objective function, which helps better identify the clustering features of complex data. The performance was compared with state-of-the-art methods in terms of different clustering measures, revealing that the proposed approach is a new clustering algorithm that can partition data with improved performance. Considering the nature of the proposed algorithm and the results of extensive experiments on various datasets, it can be applied to medical research and textual information, facilitating the extraction of critical features, and obtaining clustering results in high-dimensional and complex data conditions. The proposed algorithm significantly improves on the following aspects:

(1) The clustering result is consistent and stable, as it is not susceptible to the original cluster centers and assigns different feature weights to each cluster in the clustering process.
(2) The entropy weights improve the algorithm’s handling of partitioning during the clustering process and highlight the importance of distinguishing different features.
(3) The introduction of non-Euclidean distance makes the algorithm more robust and efficient in handling high-dimensional sparse and noisy data in the real world.
(4) The insensitivity to parameter changes ensures the flexibility of the algorithm.

In the future, EM and Gaussian mixture models will be used to improve the clustering algorithm, making it more useful in image processing. 

## Figures and Tables

**Table 1:** The computational complexity of the algorithms.

Method	Computational Complexity
K-Means	O(NKM)
WK-Means	$O(NKM^{2})$
FCM	$O(NK^{2}M)$
RLWHCM	O(NKM)
SCAD	$O(NK^{2}M+NKM^{2})$
EKM	O(NKM)
UKM	O(NKM)
Proposed algorithm	O(4NKM)

**Table 2:** Characteristics of the real-world dataset.

Dataset	Number of Samples	Number of Features	Number of Classes
Dermatology	366	34	6
Iris	150	4	3
Wine	178	13	3
Ionosphere	351	34	2
Lung career	32	56	3
Statlog (heart)	270	13	2
Zoo	101	16	7
BASEHOCK	1993	4862	2
PCMAC	1943	3289	2
ALLAML	72	7129	2
GLOMA	50	4434	4
COIL20	1440	1024	20
Yale	165	1024	15
Gisette	7000	5000	2
Madelon	2600	500	2

**Table 3:** Comparison results of the performance of algorithms on low-dimensional datasets.

Datasets		KM	WKM	FCM	RLWHCM	SCAD	EKM	UKM	Ours
Dermatology	ACC	0.36	0.41	0.36	0.68	0.47	0.53	0.36	**0.71**
	RI	0.68	0.63	0.70	0.82	0.68	0.73	0.68	0.84
	NMI	0.10	0.25	0.11	0.60	0.46	0.52	0.10	**0.64**
Iris	ACC	0.89	0.90	0.89	0.93	0.92	0.96	0.89	**0.98**
	RI	0.88	0.89	0.88	0.88	0.90	0.95	0.88	**0.97**
	NMI	0.76	0.76	0.75	0.78	0.76	0.87	0.77	**0.91**
Wine	ACC	0.70	0.70	0.69	**0.79**	0.59	0.57	0.70	0.74
	RI	0.72	0.72	0.71	**0.75**	0.62	0.54	0.72	0.73
	NMI	0.43	0.43	0.42	0.33	**0.48**	0.33	0.45	0.41
Ionosphere	ACC	0.71	0.73	0.71	0.74	0.64	0.73	0.70	**0.81**
	RI	0.59	0.63	0.59	0.66	0.54	0.54	0.58	**0.69**
	NMI	0.03	0.25	0.04	0.14	0.13	0.21	0.11	**0.27**
Lung career	ACC	0.55	0.53	0.56	0.59	0.59	0.55	0.43	**0.62**
	RI	0.58	0.63	0.63	0.55	0.56	0.59	0.46	**0.66**
	NMI	0.24	0.25	0.27	0.27	0.20	0.18	0.12	**0.29**
Statlog (heart)	ACC	0.59	0.64	0.61	0.71	0.81	0.57	0.65	**0.83**
	RI	0.51	0.54	0.52	0.61	0.70	0.51	0.52	**0.72**
	NMI	0.01	0.07	0.15	0.12	0.17	0.03	0.06	**0.23**
Zoo	ACC	0.66	0.4	0.57	0.71	0.73	0.67	0.78	**0.81**
	RI	0.81	0.23	0.83	0.83	0.75	0.80	0.85	**0.86**
	NMI	0.71	0.34	0.67	0.62	**0.77**	0.66	0.71	0.74
BASEHOCK	ACC	0.50	0.53	0.51	0.62	0.53	0.58	0.55	**0.69**
	RI	0.50	0.51	0.50	0.50	0.50	0.51	0.51	**0.55**
	NMI	0.01	0.04	0.01	0.01	0.04	0.05	0.03	**0.07**

**Table 4:** Comparison results of the performance of algorithms on high-dimensional datasets.

Datasets		KM	WKM	FCM	RLWHCM	SCAD	EKM	UKM	Ours
BASEHOCK	ACC	0.50	0.53	0.51	0.62	0.53	0.50	0.55	**0.69**
	RI	0.50	0.51	0.50	0.50	0.50	0.51	0.51	**0.55**
	NMI	0.01	0.04	0.01	0.01	0.04	0.02	0.03	**0.07**
PCMAC	ACC	0.51	0.51	0.55	0.58	0.52	0.51	0.53	**0.68**
	RI	0.49	0.50	0.50	0.53	0.59	0.57	0.58	**0.62**
	NMI	0	0.02	0.01	0.04	0.02	0.04	0.05	**0.12**
ALLAML	ACC	0.68	0.75	0.67	0.65	0.72	0.75	0.82	**0.91**
	RI	0.56	0.62	0.55	0.49	0.59	0.64	0.70	**0.84**
	NMI	0.06	0.16	0.09	0.11	0.14	0.33	0.47	**0.58**
GLIOMA	ACC	0.66	0.66	0.56	0.71	0.54	0.67	0.72	**0.76**
	RI	0.75	0.76	0.72	0.79	0.70	0.72	0.68	**0.78**
	NMI	0.48	0.55	0.56	0.59	0.53	0.57	0.52	**0.66**
COIL20	ACC	0.11	0.15	0.13	0.43	0.19	0.33	0.35	**0.41**
	RI	0.56	0.69	0.56	0.90	0.57	0.89	0.82	**0.90**
	NMI	0.28	0.40	0.29	0.58	0.21	0.42	0.49	**0.53**
Yale	ACC	0.38	0.19	0.15	0.36	0.24	0.39	0.40	**0.45**
	RI	0.88	0.47	0.58	0.88	0.76	0.84	0.87	**0.92**
	NMI	0.44	0.19	0.13	0.40	0.27	0.39	0.42	**0.55**
Gisette	ACC	0.69	0.50	0.69	0.53	0.56	0.73	0.77	**0.81**
	RI	0.57	0.50	0.58	0.51	0.52	0.62	0.66	**0.66**
	NMI	0.12	0	0.11	0	0.06	0.19	0.21	**0.26**
Madelon	ACC	0.51	0.55	0.50	0.54	0.52	0.69	0.72	**0.81**
	RI	0.50	0.50	0.50	0.52	0.5	0.62	0.65	**0.69**
	NMI	0	0.01	0	0	0.01	0.13	0.16	**0.21**

**Table 5:** Comparison results of the conditions of use of different algorithms.

Algorithm	Weight Division	Feature Constraint	Distance Division
KM	Non	Non	Euclidean
WKM	Global	Exponential	Euclidean
FCM	Non	Non	Euclidean
RLWHCM	Local	Exponential	Non-Euclidean
SCAD	Local	Exponential	Euclidean
EKM	Local	Entropy	Euclidean
UKM	Non	Entropy	Euclidean
Ours	Local	Entropy	Non-Euclidean

## Data Availability

Not applicable.
